# Encapsulation of betalain-rich extract from beetroot postharvest waste using a binary blend of gum Arabic and maltodextrin to promote a food circular bioeconomy

**DOI:** 10.3389/fnut.2023.1235372

**Published:** 2023-08-31

**Authors:** Tshamisane Mkhari, Tafadzwa Kaseke, Olaniyi Amos Fawole

**Affiliations:** ^1^Postharvest and Agroprocessing Research Centre, Department of Botany and Plant Biotechnology, University of Johannesburg, Johannesburg, South Africa; ^2^Center of Excellence for Molecular Food Sciences, Department of Biochemistry, University of Belgrade, Belgrade, Serbia

**Keywords:** freeze-drying, microencapsulation, beetroot postharvest waste, technofunctional properties, metabolites, betalain, antioxidant activity

## Abstract

**Introduction:**

The present study evaluated the potential of maltodextrin (MT), gum Arabic (GA), and their blends to produce functional beetroot waste extract powder (BWEP).

**Methods:**

The beetroot waste extracts were produced using 50% ethanol and encapsulated using 10% (1:10, *w/v*) of the GA and MT carriers at different blending ratios, namely, GA:MT 1:0, GA:MT 0:1, GA:MT 1:1, GA:MT 2:1, and GA:MT 1:2, respectively. The BWEP were analyzed for physicochemical, technofunctional, morphological, crystallinity, and antioxidant properties.

**Results:**

BWEP produced using either GA or MT exhibited better color, solubility, encapsulation efficiency, and betalain content. Powders from the blends of GA and MT showed better oil holding capacity and total phenolic content. On the other hand, powder yield, total soluble solids, titratable acidity, bulk density, and DPPH radical scavenging activity did not significantly differ (*p* > 0.05) among the powders. BWEP produced using GA and MT separately was relatively smaller and more regular compared to the powders from the blended biopolymers. All powders showed signs of agglomeration, which was more pronounced in the powders from the blended biopolymers. A total of 16 metabolites, including betalains (9), phenolic acids (2), and flavonoids (5), were tentatively identified. The majority of the metabolites were entrapped in the BWEP produced using GA and MT separately. The quantified metabolites included gallic acid (33.62–44.83 μg/g DM), (+)-catechin (32.82–35.84 μg/g DM), (−)-epicatechin (37.78–45.89 μg/g DM), and myricetin (30.07–35.84 μg/g DM), which were significantly higher in the BWEP produced from GA or MT separately.

**Discussion:**

The study showed that although blending GA and MT has the potential to improve the quality of BWEP, using these biopolymers separately showed a promise to promote a food circular bioeconomy.

## 1. Introduction

Food losses and waste persist along the value chain, resulting in an annual loss of roughly 1.3 billion tons (1). A significant portion of this waste stems from postharvest fruit and vegetable losses, ranging from 20% to 40% (2). Furthermore, these discarded fruits and vegetables are a valuable reservoir of nutrients, including vitamins A, B, and C, minerals such as calcium, potassium, and iron, and beneficial compounds like polyphenols, flavonoids, anthocyanins, tocopherols, and carotenoids (3).

The waste generated from fruit and vegetable processing is increasingly recognized as a source of these bioactive phytochemicals (4–6). This presents an opportunity for extracting and utilizing these phytonutrients in formulating functional foods and nutraceuticals. One such vegetable, beetroot, produces waste that can be turned into raw materials for valuable compounds to formulate functional foods and nutraceuticals.

Beetroot (*Beta vulgaris*) or beetroot belongs to the Chenopodiaceae family, and it is found in a variety of colors ranging from yellow to red bulbs. It is indigenous to the Mediterranean region and is grown in North America, Asia, and Europe (7). Beetroot is consumed in either a cooked or raw state. The bulk (70%) of red beetroot, which is the most common type, is processed, while approximately 30% is discarded due to poor root quality or as processing waste (7). To promote a food circular bioeconomy and reduce postharvest waste, the valorization of beetroot waste emerges as a viable option (8). Moreso, recovering bioactive phytochemicals from beetroot postharvest waste and using them to produce value-added products is a critical step toward a more sustainable food production chain (9). Despite their health importance, beetroot stems, foliage, and peels from processing are undervalued and often used as animal feed, biofertilizers, or disposed of as waste (10).

Beetroot contains a variety of health-promoting compounds, including folate, which prevents congenital disabilities; iron, which aids in the prevention and treatment of anemia; and dietary fibers, which enhance colon health (11). Moreover, beetroot is high in polyphenols, carotenoids, saponin, flavonoids, glycine betaines, betalains, and betacyanins (12). These compounds have been reported to provide beetroot with pharmacological activities such as antipyretic, anti-anemic, antihypertensive, anti-carcinogenic, anti-inflammation, anti-microbial, anti-mutagenic, anti-oxidative, anti-diabetic, anti-obesity, and cardiovascular protective effects (13). In this regard, beetroot is regarded as one of the top 10 most powerful vegetables due to its high antioxidant capacity, which is attributed to betalain compounds (14). Betalains are water-soluble nitrogenous pigments that mostly exist as red betacyanins and yellow betaxanthins. Given their hydrophilicity, non-toxicity, non-carcinogenicity, and non-poisonousness, beetroot extracts are utilized as food stabilizers or natural colorants in foods such as breakfast cereals, jam, sweets, jellies, soup, tomato pastes, and desserts (15). Nonetheless, betalains are vulnerable to environmental stresses, including pH, high temperature, light, and oxygen (16). Therefore, it is imperative to protect the betalains from these harsh environments.

Encapsulation is a widely studied technology that protects sensitive phytochemicals by forming a thin protective coating around them (17, 18). It has been demonstrated that encapsulating betalains in various edible coating matrices enhances their stability and preserves their antioxidant properties (19–22). Various encapsulation methods have been developed, including inclusion complexation, spray-drying, liposome entrapment, coacervation, co-crystallization, freeze-drying, and emulsification (23). Among these methods, freeze-drying is an effective method for drying products that are sensitive to extreme temperatures. Furthermore, it retains the product’s physical properties, such as texture, appearance, shape, and color (24). To produce a freeze-dried powder product, the use of carrier agents (biopolymers) is necessary.

Maltodextrin (MD) and gum Arabic (GA) are the most commonly used biopolymers in producing freeze-dried powder products due to their high solubility, biocompatibility, optimum viscosity, and stability (25, 26). MD, for instance, has been successfully employed to encapsulate bayberry polyphenols (27), pomegranate peel extract (28), casein hydrolysates (29), and sumac extract (30). Meanwhile, GA alone or combined with MD has also been utilized to encapsulate saffron petal anthocyanins (31), pomegranate juice (32), grape polyphenols (26), mountain tea water extract (33), and sour cherry juice (34). These findings revealed that blending GA and MD significantly improved the physicochemical properties of the developed powders, and this was ascribed to the varied properties of these two biopolymers.

In the context of encapsulation of beetroot and its waste, although some research exists (17, 21, 22), the scientific literature remains noticeably deficient in studies exploring the use of binary blends of GA and MD for the encapsulation of beetroot waste. Therefore, the present study aimed to investigate the effect of blending GA and MD using beetroot postharvest waste at varied proportions on the physicochemical, technofunctional, morphological and antioxidant properties of encapsulated beetroot waste extract powder for potential utilization in the food industry.

## 2. Materials and methods

### 2.1. Plant material and chemicals

The selection of beetroot for our study was based on its unsuitability for fresh consumption due to cosmetic flaws such as excessive shrinkage and mechanical damages such as blemishes, bruises and cracks. Such low-quality beetroot is regarded as postharvest waste. Samples were collected from the Johannesburg Farmers Market, Gauteng Province, South Africa, and delivered to the University of Johannesburg’s Postharvest Research Laboratory. The beetroot was thoroughly washed in 1% acetic acid, cut into thin slices, oven-dried at 40°C ± 2°C for 12 h to dry to a constant weight. Chemicals including gum Arabic, maltodextrin, Folin-Ciocalteau, gallic acid, 6-hydroxy-2,5,7,8-tetramethylchroman-2-carboxylic acid, 2,2-diphenyl-1-picrylhydrazyl, catechin, ellagic acid, epicatechin, gallic acid, punicalagin, punicalagin and chlorogenic acid, rutin, syringic acid, and quercetin, were all of analytical grade and were purchased from Merck, South Africa.

### 2.2. Preparation of extracts from beetroot waste powder

Beetroot waste powder (25 g) was mixed with 50% aqueous ethanol (250 mL) containing 0.5% and then ultrasonicated at room temperature for 30 min at maximum power (700 W) and frequency (40 kHz) using a Labotec ultrasound bath (Johannesburg, Gauteng Province, South Africa). The mixture was filtered using a 47 mm diameter Whatman filter paper and concentrated under reduced pressure (150 mbar) using a BUCHI Rotavapor R-300 (Flawil, Switzerland) at 40°C ± 2°C until all the ethanol was removed.

### 2.3. Encapsulation and freeze-drying procedure

At room temperature (25°C ± 2°C), beetroot wastes extract was mixed with 10% (w/v) GA and MT blends [GA:MT (0:1), GA:MT (1:0), GA:MT (1:1), GA:MT (1:2), and GA:MT (2:1)] using a magnetic stirrer. The blended samples were then homogenized for 45 s using a Stuart SHM2 ultra-homogenizer (Staffordshire, United Kingdom) before they were frozen for 24 h at −20°C and freeze-dried for 48 h using a Buchi Lyovapor L-200 Freeze Dryer (Postfach, CH-9230, Flawil, Switzerland) at 60°C and 0.01 millibar. The freeze-dried samples were ground into a fine powder (<1 mm particle size). The beetroot waste extract powder (BWEP) yield was calculated as grams of powder per 100 g of total solids in the feed solution and reported as a percentage (%).

### 2.4. Analysis of the physicochemical properties of beetroot waste extract powder

#### 2.4.1. Moisture content and color

The moisture content (MC) of the beetroot waste extract powder (BWEP) was measured using a moisture analyzer (KERN DBS 60-3, Berlin, Germany) set at 100°C. A calibrated chromometer (CR-10 Plus, Konica Minolta, Osaka, Japan) was used to measure the color attributes, including lightness (L*), redness (a*), and yellowness (b*). Respectively, Chroma (C*), hue angle (h°), and total color differences (∆E) were calculated using Equations 1, 2. All measurements were done in triplicates.

(1) $$\Delta E={\left[{\left(\Delta L^{*}\right)}^{2}+{\left(\Delta a^{*}\right)}^{2}+{\left(\Delta b^{*}\right)}^{2}\right]}^{1/2}$$

(2) $$C^{*}={\left(a^{*2}+b^{*2}\right)}^{1/2}$$

where ∆L, ∆a*, and ∆b* represents changes in lightness, redness, and yellowness/blueness, respectively.

#### 2.4.2. Titratable acidity, total soluble solids, and pH

BWEP (25 g) was dissolved in 2.5 mL distilled water, vortexed for 5 min, and then sonicated using a Sonic ultrasound bath (Labotec, Johannesburg, Gauteng Province, South Africa). The samples were then centrifuged at 8,400 x g for 25 min (Thermo Fischer Scientific, Biofuge, Stratos, United Kingdom), and the supernatant obtained was used to determine titratable acidity (TA), total soluble solids (TSS), and pH. TA was measured using an auto-titrator (Orion Star T910, Thermo Fisher Scientific, Sussex, United Kingdom). Briefly, the supernatant (2 mL) was mixed with 90 mL of deionized water and then immediately titrated against sodium hydroxide (0.2 N) until the pH was 8.2. The results were presented as a percentage. An ATAGO PT-32 refractometer (Tokyo, Japan) was employed to determine TSS, and the results were reported in °Brix. The pH was measured using an Insmark LS128 pH meter (Mumbai, India). All measurements were done in triplicates.

### 2.5. Analysis of the technofunctional properties of beetroot waste extract powder

#### 2.5.1. Bulk density and hygroscopicity

To determine bulk density, BWEP (2 g) was put in a 10 mL cylinder, and the cylinder was dropped into a polystyrene container 10 times from a height of 15 cm. The bulk density was calculated by dividing the powder’s mass by its volume (35). The method described by Adetoro et al. (36) was slightly modified to measure hygroscopicity. In brief, 1 g of the beetroot waste extract powder was put in a desiccator with a saturated solution of sodium chloride (75% RH). After 24 h, the samples were weighed, and hygroscopicity was expressed as grams of absorbed moisture per 100 g of dry powder. All measurements were done in triplicates.

#### 2.5.2. Solubility, oil, and water holding capacity

Deionized water (50 mL) was added to BWEP (0.5 g), and the mixture was vortexed for 30 s and centrifuged for 5 min at 756 × g and 4°C using a Thermo Fisher Scientific Biofuge centrifuge (Stratos, United Kingdom). After that, the supernatant (12.5 mL) was transferred to a pre-weighed petri dish and dried for 5 h in a 105°C oven. The difference in weight before and after drying was used to calculate the solubility, which was expressed as a percentage. The oil-holding capacity (OHC) and water-holding capacity (WHC) were determined according to a previous method (35). All measurements were done in triplicates.

### 2.6. Analysis of total phenolic content, betalain content, encapsulation efficiency, and radical scavenging activity of the beetroot waste extract powder

#### 2.6.1. Preparation of extracts from encapsulated beetroot waste

Triplicate samples of beetroot waste extract powder (0.5 g) were separately mixed with 10 mL of 50% methanol, vortexed for 30 s, sonicated for 2 min, and centrifuged (Thermo Fischer Scientific, Biofuge, Stratos, United Kingdom) at 5,590 × g for 5 min. The supernatants were used to determine the total phenolic content (TPC) and the radical scavenging activity. All measurements were done in triplicates.

#### 2.6.2. Total phenolic content, betalain content, and encapsulation efficiency

The methods described by Sarabandi et al. (37) and Magangana et al. (38) were used to determine total phenolic content (TPC). The beetroot waste extract (50 μL), 50% methanol (450 μL), and Folin-Ciocalteau (500 μL) were mixed in a cuvette and then incubated in the dark for 10 min at room temperature. Then, 2.5 mL of 2% sodium carbonate solution was added, and the samples were further incubated for 40 min. The absorbance measurements were taken at 750 nm using a UV visible spectrophotometer (SP-UV 300, Shanghai, China). Gallic acid was used to prepare the standard curve (0.01–20 g/mL, R^2^ = 0.9825), and the TPC results were expressed as mg GAE /100 g powder. All measurements were done in triplicates.

The betalain content was determined following the method of Fawole et al. (39). The BWEP was dissolved in distilled water, filtered, and the absorbance of the filtrate measured at 538 nm using a UV visible spectrophotometer (SP-UV 300, Shanghai, China). The total betalain content was calculated using Equation 3.

(3) $$\mathrm{BC (mg/100g)}=\frac{A\times \mathrm{DF}\times \mathrm{MW}\times V\times 100}{\varepsilon \times L\times W}$$

where BC is the betanin content (mg/100 g), A is the absorbance at 538 nm, DF is the dilution factor, MW is the betanin molecular weight (550 g/mol), V is the pigment solution volume (mL), ε is the betanin molar extinction coefficient (60,000 L/mol/cm), L is the cuvette path length (1 cm), and W is the weight of encapsulated beetroot waste extract powder (g).

The betalain encapsulation efficiency (BEE) was calculated as the ratio between the betalain content in the beetroot waste extract powder and the initial infeed solutions using Equation 4.

(4) $$\begin{array}{l} \mathrm{BEE}\;\left(\%\right)=\\ \frac{\mathrm{Betalain content in encapsulated betroot waste powder}}{\mathrm{Betalain content in infeed solution}}\times 100 \end{array}$$

#### 2.6.3. DPPH radical scavenging activity

The reaction mixture contained 15 μL beetroot waste extract, 735 μL of 50% v/v methanol, and 750 μL of a 0.1 mM methanolic DPPH solution (2). The samples were incubated for 30 min in the dark before their absorbances were measured at 517 nm using an SP-UV 300 UV–vis spectrophotometer (Shanghai, China). The standard curve was prepared using Trolox (0–2 mM; R^2^ = 0.9990), and the results were expressed as mM Trolox equivalent per gram powder (mM TE/g powder). All measurements were taken three times.

### 2.7. Microstructure analysis of the beetroot waste extract powder

The BWEP particles were analyzed using a scanning electronic microscope (SEM; Tescan Vega 3, Borno, Czech Republic). The samples were held together with adhesive tape before being coated with a fine layer of gold and examined at 100 and 500× magnifications. The images were processed using the ImageJ software (National Institutes of Health, Bethesda, MD, United States).

### 2.8. LCMS analysis of secondary metabolites in the beetroot waste extract powder

The individual phenolic compounds in the BWEP were analyzed using liquid chromatography-mass spectrometry (LC-MS/MS) following a previously described method (37). A Waters Synapt G2 quadrupole time-of-flight mass spectrometer (Milford, MA, United States) was used for profiling, along with a Waters Acquity UPLC and a Waters HSS T3 column (2.1 × 100 mm, 1.7 m). The flow rate and injection volume of the solvent A (0.1% formic acid) and solvent B (0.1% acetonitrile) mobile phases were 0.3 mL/min and 2 L, respectively. The gradient elution followed the conditions: 0 min: 100% A, 1–22 min: 28% B, 50 s: 40% B, and 1.5 min: 100% B. This was further re-equilibrated for 5 min. A stock solution containing pure standards (0.1–50 mg/mL), catechin, ellagic acid, epicatechin, gallic acid, punicalagin, punicalagin and chlorogenic acid, rutin, syringic acid, and quercetin was used to determine the structures and quantitative analyses of the phenolic compounds. This was done using calibration curves and the structure-related target analyte/standard (chemical structure and or functional group) principle. For the regression coefficient, good linearity (R^2^ > 0.990) was obtained. The MassLynx 4.1 software was used to collect and process the data, and a metabolomic method was employed to highlight the significant and minute differences and similarities.

### 2.9. Statistical analysis

The results of the triplicate samples are presented as a mean ± SE (standard error). Data were analyzed using the STATISTICA software (STATISTICA v13, TIBC, Palo Alto, CA 94304, United States) and SAS software (SAS Enterprise Guideline 7.1, SAS Enterprise, Carrey, NC, United States) and one-way analysis of variance (ANOVA). The means were separated using Duncan’s multiple range test at a 5% significance level.

## 3. Results and discussion

### 3.1. Yield, moisture content, and color attributes

Table 1 presents the yield, MC, and color attributes of the BWEP. The powder production yield in the present study varied from 14.83%–15.30%, and no significant variation (*p* > 0.05) was found among the BWEP samples. These findings suggest that blending GA and MT did not impact the powder production yield, irrespective of the different biopolymer ratios. These results contrast with some previous findings. For instance, Jafari et al. (26) observed that spray dried grape polyphenol extract powders produced using MT or GA separately showed higher yield than powders produced using their blends. Similar findings were also reported by Adetoro et al. (36) from GA and MT-encapsulated and spray dried eggplant peel extract powders. This discrepancy between our results and those in the existing literature could be due to differences in plant extract types and drying techniques. Given the potential applications of BWEP within the food industry, future studies should explore strategies to enhance powder yield.

**Table 1:** Yield (%), moisture content (%), and color attributes of freeze-dried BWEP developed using different blends of gum Arabic and maltodextrin.

Carrier	Yield	MC	L*	a*	b*	C*	∆E
GA:MT (1:0)	14.83 ± 0.57^a^	4.99 ± 0.38^a^	31.09 ± 0.65^c^	51.58 ± 0.71^b^	10.74 ± 0.84^d^	167.04 ± 19.09^d^	696.64 ± 37.48^b^
GA:MT (0:1)	15.20 ± 0.69^a^	3.27 ± 049^b^	39.10 ± 0.60^a^	54.08 ± 0.73^a^	19.59 ± 0.53^a^	437.90 ± 21.39^a^	994.44 ± 66.18^a^
GA:MT (1:1)	15.30 ± 0.10^a^	4.05 ± 0.10^a^	33.85 ± 1.23^b^	50.30 ± 0.15^c^	6.89 ± 0.26^e^	97.78 ± 3.79^e^	616.75 ± 9.41^c^
GA:MT (1:2)	15.10 ± 0.35^a^	3,21 ± 0.81^b^	34.13 ± 0.54^b^	48.97 ± 0.84^d^	14.74 ± 0.60^b^	266.58 ± 17.34^b^	622.86 ± 35.91^bc^
GA:MT (2:1)	14.90 ± 0.62^a^	3.97 ± 0.21^a^	33.18 ± 0.38^b^	50.70 ± 0.67^bc^	13.14 ± 0.91^c^	231.27 ± 23.34^c^	685.25 ± 20.29^bc^

Values represent the mean ± SE of triplicate tests (*n* = 3). Values in rows with different superscripts are significantly different (*p* < 0.05) according to Duncan’s multiple range test. GA, gum Arabic; MT, maltodextrin; MC, moisture content; L*, lightness, a*, redness; h°, hue angle; C*, chroma; ∆E, total color difference.

MC significantly impacts a powder’s technofunctional properties and chemical and microbiological stability (40). The MC of the BWEP ranged from 3.21–4.99%. Such a range is generally considered safe to inhibit microbial growth and biochemical reactions, allowing for extended storage periods without MC-related degradation (40). The MC reported in the current study is within the range reported by Dag et al. (41) for GA and MT encapsulated beetroot juice. A significantly lower MC was noted in BWEP encapsulated powders when MT was used alone or when its concentration was twice that of GA (Table 1). However, using GA alone or increasing its concentration did not significantly (*p* > 0.05) alter the powders’ MC. The higher MC in GA encapsulated powders compared to MT encapsulated powders could be due to the larger proportion of hydrophilic groups in GA (42, 43). This finding aligns with the observations reported by Mohd Nawi et al. (44) for encapsulated *Moringa oleifera* leaf extract using GA, MT, and their blends.

**Figure 1:**
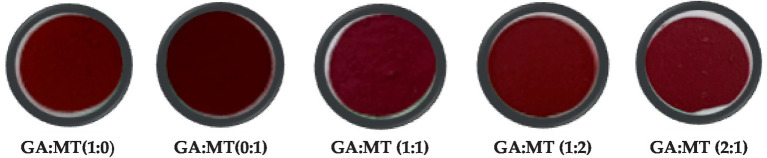
Color images of freeze-dried beetroot waste extract powder developed using different blends of gum Arabic (GA) and maltodextrin (MT).

Consumer’s decision to buy food products primarily depends on physical attributes such as color (45). Table 1 presents the color parameters L*, a*, b*, C*, and ∆E of BWEP, while Figure 1 shows images of the powders. All BWEP showed a positive a* due to the natural red color of beetroot, linked to a high concentration of betacyanin content. BWEP produced using MT alone exhibited the highest a*. Blending GA and MT significantly reduced the a* of BWEP, with GA:MT (1:2) exhibiting the lowest a*. The visually discernible color distinctions of beetroot waste extract powder support these findings (Figure 1). Powders produced using MT alone showed the highest L* (39.10). Higher L* values were also reported in MT-encapsulated raspberry powder (2), passion fruit peel (46), and mango juice powders (47) when compared to the same powders produced using GA. However, blending GA and MT significantly increased the L* compared to using GA alone. Powders produced using MT alone and increased concentration of MT exhibited higher C*. Similarly, the highest ∆E was observed in BWEP produced using MT alone (Table 1). Blending the carrier agents at different proportions did not significantly (*p* > 0.05) affect the ∆E. This observation follows a similar trend reported by Barbosa et al. (48). These color results suggest a higher concentration of betacyanin content in BWEP produced using MT alone relative to GA and powders from the blended carriers.

### 3.2. Total soluble solids, titratable acidity, and pH

According to Table 2, the lowest (9.02 °Brix) and highest (9.43 °Brix) TSS were observed in GA:MT (0:1) and GA:MT (1:2), respectively. The TSS from the other samples did not significantly differ (*p* > 0.05). The TA for GA and MT were 0.150% and 0.170%, respectively, and significantly differed between these samples. However, blending GA and MT did not significantly affect the TA (Table 2). The TSS and TA results indicate that the carrier agents did not affect the beetroot’s soluble compounds or organic acids. Given that TSS and TA are positively linked to the nutritional quality and taste of foods, the observed results suggest that the BWEP are suitable for incorporation into different food product formulations. The pH of beetroot waste extract powder is crucial for regulating the availability of nutrients, microbial activity, and biological functions. The pH of BWEP ranged between 4.73 and 4.84 and varied insignificantly among the samples. These low pH values are desirable to prevent microbial growth and promote the stability of betalains during storage (49). The pH results corroborate the TA results.

**Table 2:** Physicochemical and technofunctional properties of freeze-dried BWEP developed using different blends of gum Arabic and maltodextrin.

Carrier	TSS (°Brix)	TA (%)	pH	Solubility (%)	BD (g/cm^3^)	Hygroscopicity (%)	WHC (%)	OHC (%)
GA:MT (1:0)	9.10 ± 0.02^ab^	0.150 ± 0.014^b^	4.84 ± 0.02^ab^	51.00 ± 3.61^b^	0.67 ± 0.01^a^	1.33 ± 0.06^c^	0.44 ± 0.05^a^	0.25 ± 0.04^b^
GA:MT (0:1)	9.02 ± 0.00^b^	0.170 ± 0.010^a^	4.73 ± 0.08^b^	60.33 ± 2.52^a^	0.68 ± 0.03^a^	4.13 ± 0.06^a^	0.40 ± 0.04^ab^	0.26 ± 0.03^b^
GA:MT (1:1)	9.17 ± 0.23^ab^	0.165 ± 0.002^ab^	4.84 ± 0.12^ab^	34.33 ± 1.53^c^	0.66 ± 0.01^a^	2.80 ± 0.36^b^	0.36 ± 0.04^b^	0.24 ± 0.04^b^
GA:MT (1:2)	9.43 ± 0.15^a^	0.161 ± 0.004^ab^	4.84 ± 0.05^ab^	36.00 ± 2.00^c^	0.74 ± 0.03^a^	1.27 ± 0.12^c^	0.41 ± 0.03^ab^	0.44 ± 0.02^a^
GA:MT (2:1)	9.07 ± 0.06^ab^	0.156 ± 0.04^ab^	4.88 ± 0.04^a^	48.33 ± 2.08^b^	0.71 ± 0.07^a^	1.43 ± 0.23^c^	0.47 ± 0.06^a^	0.50 ± 0.03^a^

Values represent the means of replicates tests (*n* = 3). Different superscripts in each row indicate significant differences (*p* < 0.05) according to Duncan’s multiple range test. GA, gum Arabic; MT, maltodextrin; TSS, total soluble solids; TA, titratable acidity; BD, bulk density; WHC, water-holding capacity; OHC, oil-holding capacity.

### 3.3. Solubility, bulk density, and hygroscopicity

Powder solubility is an important quality metric to assess its ability to disintegrate in solutions. The solubility of BWEP produced in the present study varied from 34.33%–51.00% (Table 2). The low solubility of BWEP could be explained by the decrease in hydrophilic groups available to bind water due to the linkages with polyphenols, betalains, betacyanins, and betaxanthins (17). However, these results were higher than those reported from freeze-dried betabel extract (*Beta vulgaris*) powder encapsulated with MT and inulin (17). Overall, the solubility of individual powders statistically varied significantly from that of blended powders (*p* < 0.05). Blending the two carriers significantly decreased the BWEP solubility by 1.1–1.8 fold, a phenomenon that is attributable to intermolecular and chemical interactions. The higher solubility of BWEP produced using MT alone (60.33%) compared to the rest of the powders could be due to the biopolymer’s short and branched chains with high hydrophilicity (50). This indicates that MT-encapsulated BWEP can be easily integrated and distributed into other products during product development. The MT microcapsules have been previously reported to exhibit higher solubility (33, 36, 51, 52).

Bulk density can be used to evaluate a powder’s ease of storage, packing, and transportation. The BWEP bulk density in the current study (0.66–0.74 g/cm^3^) exhibited no significant difference (*p* > 0.05), implying that the bulk density was independent of the carrier agents. It is noteworthy that the bulk density of BWEP in this study was higher than that of eggplant peel powders (0.50–0.58 g/cm^3^) (36) and beetroot juice powders [0.512–0.549 g/mL; (48)] and whey protein powder [0.527–0.546 g/mL; (41)], all of which were encapsulated using MT and GA. The relatively high bulk density of BWEP signifies its compactness, implying that it would require less storage space, making it easier to handle. Furthermore, higher bulk densities are often associated with low air content in microcapsules. Low air content minimizes the risk of air-related degradation processes, such as oxidation, suggesting that the BWEP, with its higher bulk density, could exhibit enhanced resistance against oxidative degradation during storage.

Hygroscopicity determines the ability of a powder to absorb moisture in response to ambient humidity and is implicated in the storage stability of powders (45). The hygroscopicity of the various microcapsule samples ranged from 1.27% to 4.13% (Table 2). According to the GEA Niro, there are three types of powder hygroscopicity: hygroscopic (15.1%–20%), mildly hygroscopic (15.1%–20%), and non-hygroscopic powder (<10%). This categorization classifies all BWEP as non-hygroscopic. This implies that the powders could be shelf-stable for a prolonged period. The highest hygroscopicity was observed in powders produced using MT alone (4.13%), and the lowest hygroscopicity was observed in GA:MT (1:2; 1.27%) powders. When the GA and MT were blended, the hygroscopicity significantly decreased compared to powders produced using MT alone. As stated earlier, the intermolecular and chemical interactions could have reduced the hydrophilic groups available to interact with the water molecules in the environment. The hygroscopicity values from the present study are lower than those reported in the literature (41, 53).

### 3.4. Water holding capacity and oil holding capacity

The water holding capacity (WHC) of powders is essential in food formulation as it determines how a product will interact with water. In this study, the WHC of the powders was not significantly influenced (*p* > 0.05) by blends or the individual carriers (Table 2). This suggests that the ability of the powders to retain water remained constant, irrespective of the differences in compositions and configurations of GA, MT, and their blends. This contrasts with findings from a study on encapsulated passion fruit extracts where MT-encapsulated powder showed a higher WHC than GA-encapsulated powder (46). Given the variance in morphology among our powder samples, interpreting the WHC findings should be done carefully.

On the other hand, the oil holding capacity (OHC) of the powders was highest in samples produced by blending GA and MT at ratios of 1:2 and 2:1 (Table 2). This suggests that doubling the concentrations of GA and MT might have enhanced the powders’ hydrophobicity, thus increasing their capacity to interact with lipids. Comparatively, the OHC results from this study were lower than those of GA and MT encapsulated tomarillo juice powder reported by Dobroslavić et al. (54). This discrepancy stresses the influence of raw materials and encapsulating agents’ unique properties on the final product characteristics.

### 3.5. Betalain content, total phenolic content, betalain encapsulation efficiency, and DPPH radical scavenging activity

**Figure 2:**
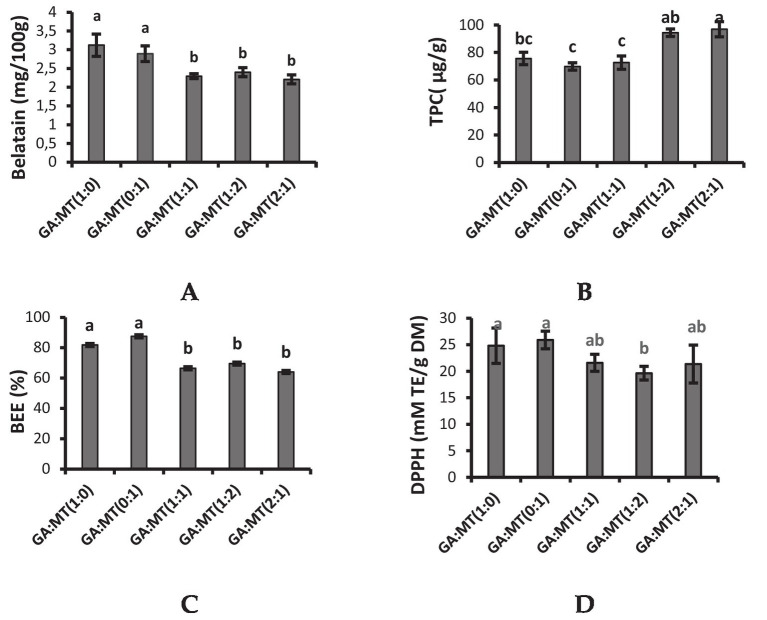
Phytochemicals and antioxidant activity of beetroot waste extract powders encapsulated using different blends of GA and MT. **(A)** Betalain content, **(B)** Total phenolic content (TPC), **(C)** Betalain encapsulation efficiency (BEE), **(D)** DPPH radical scavenging activity. The different letters on the vertical bars represent significant differences in means (*p* < 0.05) and number of replicates (*n* = 3) according to Duncan’s multiple range test. Vertical bars on each bar indicate standard deviation of the mean. GA, gum-Arabic; MT, maltodextrin.

Beetroot contains betalain, a secondary plant metabolite responsible for its red color and biological activities. The betalain content of BWEP produced in the present study varied from 2.2–3.12 mg/100 g (Figure 2A) and was lower than the betalain content of GA and MT-encapsulated beetroot powders reported in the literature (17, 21, 41). The variation in betalain content could be ascribed to cultivar., harvest maturity, encapsulation method, and extraction method differences, among other factors. A higher betalain concentration was observed in powders produced using GA or MT alone (Figure 2A). According to Dag et al. (41), proteins have good emulsification properties and could have acted as wall material to prevent oxidative damage during freeze-drying. Combining the two carriers drastically decreased the betalain content by 17%–28%, which did not significantly (*p* > 0.05) vary among the powders from the carriers’ blends. Blending GA and MT could have affected the hydrophilic groups available to interact with the betalain, thereby affecting its encapsulation.

Reactive oxygen species scavenging, electrophile scavenging, and metal chelation are some of the antioxidant properties of phenolic compounds. TPC is thus one of the crucial indices used to evaluate the antioxidant activity of different plant extracts. The TPC of the powders in the present study varied from 69.78–96.90 μg/g and was lower than the TPC results reported from previous studies on GA and MT-encapsulated beetroot juice powder (17, 48). Blending GA and MT significantly improved the TPC, especially for the powders produced when the concentration of either GA or MT was doubled (Figure 2B). This observation was comparable to the results reported by Barbosa et al. (48) from beetroot juice powder produced from blends of GA and MT. However, Ramakrishnan et al. (55) reported higher TPC in mulberry powders produced using GA, MT, and whey proteins alone than in the carriers’ blends. The finding that BWEP produced from blending GA and MT showed higher TPC contradicts the TPC encapsulation efficiency results (Figure 2B), which were lower in the respective powder samples. Therefore, blending GA and MT could have caused an overestimation of the TPC in the powders due to the presence of compounds such as sugars, which could absorb at the same wavelength as polyphenols.

Betalain encapsulation efficiency (BEE) refers to a wall material’s capacity to keep the core substance contained within the microcapsule. BEE of the freeze-drying process was calculated from the TPC of the initial extract. While the BEE of BWEP produced by GA and MT individually ranged from 82 to 88%, the combination of the carrier agents decreased the BEE of the BWEP (64%–70%) (Figure 2C). These findings imply that MT and GA blending reduced the capacity of the polyphenols to bind to the biopolymers. MT has been reported to confer a protective effect on the encapsulated compounds, and this could explain the slightly higher BEE observed in BWEP produced using MT alone (56). Although the literature has reported that a combination of wall materials improves the BEE of the targeted compounds, blending GA and MT had a negative effect on BEE, suggesting that other blending options should be studied.

To ascertain if the encapsulating process impacted the antioxidant qualities of the beetroot’s bioactive components incorporated in the microcapsules, the antioxidant activity of BWEP was studied using the DPPH assay. As seen in Figure 2D, there were no significant differences (*p* > 0.05) in the DPPH radical-scavenging activity (*p* > 0.05) among most of the powders, except for GA:MT (1:2), which showed a significantly lower DPPH radical scavenging activity. Insignificant variation (*p* > 0.05) in DPPH radical scavenging activity was also reported by Seerangurayar et al. (46) in GA and MT-encapsulated passion fruit peel extract powders. However, Barbosa et al. (48) reported higher DPPH radical scavenging activity in beetroot powders produced using the blends of GA and MT than in powders produced using the biopolymers individually. These results were attributed to the good emulsifying properties of GA and the oxidation-protective effect of MT. Cano-Chauca et al. (53) also showed that combinations of carriers have the potential to improve the powder’s antioxidant capacity. The variation in the antioxidant activity of the GA and MT powders from the different studies could be explained by the complexity of the phytochemicals in plant extracts and their differences in interacting with the biopolymers. The DPPH radical scavenging activity results exhibited the same trend as that of betalain content, suggesting that betalains could be the main compounds contributing to the antioxidant activity of beetroot extracts.

### 3.6. Surface morphology of BWEP

**Figure 3:**
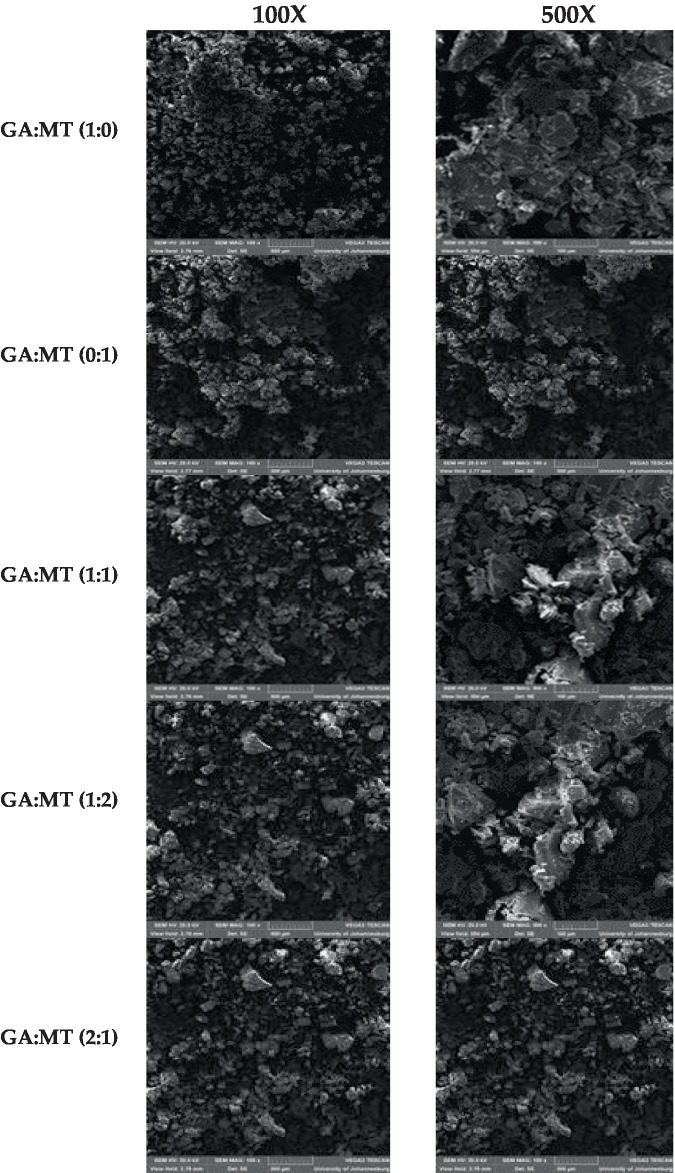
Scanning electron microscopy micrographs of freeze-dried beetroot waste extract powder produced using different blending ratios of gum Arabic (GA) and maltodextrin (MT).

The surface morphology of powders greatly affects their technofunctional properties, such as bulk density, compaction, flowability, solubility, and rehydration, which are primarily dictated by particle size and shape. Figure 3 illustrates the distinct surface structures of the powders formulated with GA and MT, and their blends. Powders prepared with GA or MT alone exhibited different external morphologies than those formulated with their blends, as seen under 100 and 500× magnification. Powders produced with GA or MT displayed smaller, regular particle sizes with smooth surfaces. Conversely, blending GA and MT resulted in powders with relatively larger, irregular particles featuring flaky and rough surfaces. According to Mohd Nawi et al. (44), powders with large and irregular particles may demonstrate poor storage stability due to increased exposure to oxidative degradation, indicating a need for further studies on storage capabilities. Particle agglomeration was observed in all powder samples but was more pronounced in the powders from the GA and MT blends. This could explain the low solubility observed in all powder samples (Table 2). Agglomeration, a typical feature of freeze-dried powders, has been linked to static electricity effects and Van der Waals forces; however, exposed hydrophilic groups in GA and MT may also be implicated. According to the literature, agglomeration may enhance powder stability (46). To ascertain this phenomenon, further studies are needed, given that powder samples that exhibited more agglomeration also showed bigger and irregular particles, which may negatively impact their storage stability. Crystals were observed on all powders; however, they were more prominent in the powders produced using the blends of GA and MT. GA:MT (1:1) and GA:MT (1:2) showed more visible evidence of crystal formation, especially at 500× magnification. No evidence of cracking was observed in all the powder samples, a development that is vital in preventing oxidative degradation of the encapsulated bioactive phytochemicals (46).

### 3.7. Metabolomic analysis of the beetroot waste extract powder

#### 3.7.1. Tentative identification of metabolites

Betalains (9), phenolic acids (2), and flavonoids (5) were among the 16 compounds that were tentatively identified and characterized in the BWEP. Literature has highlighted these compounds as the main secondary metabolites in beetroot (57). Betalains, which are water-soluble nitrogen-containing pigments that provide red-violet and yellow (betacyanins and betaxanthins, respectively) colors to vegetables and fruits (ca. 41% of the total), were the primary compounds identified in the BWEP (Table 3). These included betanin (compound 4, RT = 4.42 min, m/z 551.1517), neobetanin (compound 6, RT = 4.57 min, m/z 549.116), isobetanin (compound 7, RT = 4.72 min, m/z 551.1587), portulaxanthin (compound 1, RT = 1.08 min, m/z 272.9551), and their derivatives, which included iso-vulgaxanthin IV isomer (isoleucine-iso-bx; compound 2, RT = 1.50 min, m/z 325.1160), 2′-apiosyl-betanin (compound 3, RT = 1.69 min, m/z 683.2228), 17-decarboxy-neobetanin (compound 5, RT = 4.47 min, m/z 505.1461), vulgaxanthin IV isomer (isoleucine-bx; compound 8, RT = 6.64 min, m/z 325.1411), and dehydrogenated tridecarboxy-neophyllocactin (compound 9, RT = 6.72 min, m/z 501.1149). Since betalains are commercially recognized as natural food colorants with health-promoting properties, their abundance in the BWEP is encouraging. These compounds provide beetroot with anticancer properties, hepatic protective effect, and protection against peroxidation and DNA damage in cells (57). Phenolic acids were also tentatively identified in the BWEP. The two phenolic acids (ca. 9% of the total) were gallic acid (compound 10, RT = 5.02 min) and ferulic acid 4-glucoside (compound 11, RT = 8.25 min) with observed m/z of 169.0146, and 355.2574, respectively. Four flavonoid compounds (ca. 18% of the total) which included (+)-Catechin (compound 12, RT = 8.51 min, m/z 289.0733), (−)-Epicatechin (compound 13, RT = 8.75, m/z 289.8355), myricetin (compound 14, RT = 11.51, m/z 317.8787), and betagarin (compound 15, RT = 12.42, m/z 329.2297) were also tentatively identified in al the powder samples. However, the current study could not identify seven compounds (Unknowns A-F) with retention times ranging from 1.24–14.42 min and m/z values varying from 147.0774 to 955.4524 (Table 3).

**Table 3:** List of compounds tentatively identified in beetroot waste extract powders showing retention times, detected by both ESI positive and negative ionization mode elemental composition, MS^E^ fragments, and UV absorbance.

No.	Rt (min)	Experimental *m/z* [M + H]^+^ /[M − H]^−^		MS^E^ Fragments formula	Elemental	UV (nm)	Tentative Identity	GA:MT (1:0)	GA:MT (0:1)	GA:MT (1:1)	GA:MT (1:2)	GA:MT (2:1)
1	1.08	272.9551	[M − H]^−^	**158.9789,** 272.9635, 288.9378, 114.9882	C_5_H_5_O_13_	224	Portulaxanthin	+	+	+	+	+
2	1.50	325.1160	Both	**325.1456,** 149.0232, 99.0440	C_15_H_20_N_2_O_6_	220	Iso-vulgaxanthin IV isomer (isoleucine-iso-bx)	+	+	+	+	+
3	1.69	683.2218	[M − H]^−^	**341.1114,** 683.2335, 179.0582	C_12_H_21_O_11_	227; 468	2′-apiosyl-betanin	+	+	+	+	+
4	4.42	551.1517	Both	**389.1043,** 551.1603	C_24_H_27_N_2_O_13_	220; 466	Betanin	+	+	+	+	+
5	4.47	505.1461	[M − H]^−^	**194.0460,** 356.1002, 505.1454, 255.21134, 150.0555	C_23_H_24_N_2_O_11_	220; 468	17-decarboxy-neobetanin	+	+	+	+	+
6	4.57	549.1559	[M − H]^−^	**549.1231,** 505.4656, 345.5656, 150.6766	C_24_H_24_N_4_O_13_		Neobetanin	+	+	+	+	+
7	4.72	551.1587	Both	**389.1065,** 551.1567	C_24_H_27_N_2_O_13_	220; 472	Isobetanin	+	+	+	+	+
8	6.64	325.1411	Both	**325.2326**, 86.0969	C_15_H_20_N_2_O_6_	222	Vulgaxanthin IV isomer (isoleucine-bx)	+	+	+	+	+
9	6.72	501.1149.	[M − H]^−^	**251.0820,** 501.1113, 517.1636, 295.0716	−	220; 426	Dehydrogenatedtridecarboxy-neophyllocactin	+	+	+	+	+
10	5.02	169.0146	[M − H]^−^	**169.014,** 125.025, 124.017, 89.5455	C_7_H_5_O_5_	270; 259	Gallic acid*	+	+	+	+	+
11	8.25	355.2574	[M + H]^+^	**355.5645,** 187.9767, 89.0009	−	220	Ferulic acid 4-glucoside	+	−	−	−	−
12	8.51	289.0733	[M-H]^−^	**289.0791,** 245.4082, 203.5072, 109.0728	C_15_H_13_O_6_	270, 259	(+)-Catechin^*^	+	+	+	+	+
13	8.75	289.8355	[M-H]^−^	**289.9776,** 236.7655, 203.7555, 89.5655	C_15_H_13_O_6_	270, 259	(−)-Epicatechin^*^	+	+	+	+	+
14	11.51	317.8787	[M − H]^−^	**317.8767,** 151.5455, 178.4338, 89.6565	C_15_H_10_O_8_	372	Myricetin^*^	+	+	+	+	+
15	12.42	329.2297	[M − H]^−^	**329.2258**, 171.0948	C_18_H_33_O_5_	220;458	Betagarin	+	+	+	+	+
16	1.24	147.0774	[M + H]^+^	**147.9888,** 238.6566, 89.5655	−	220	Unknown A	+	+	+	+	+
17	3.82	208.0200	[M − H]^−^	**208.6766**, 168.7677, 89.9980	−	220	Unknown B	+	+	+	+	+
18	5.43	310.0932	[M − H]^−^	**163.0397,** 310.0930, 89.0978	C_9_H_8_O_3_	220; 296	Unknown C	+	+	+	+	+
19	9.18	187.0974	[M − H]^−^	**187.0984**, 209.0789, 225.0492, 125.0940	C_9_H_15_O_4_	220	Unknown D	+	+	+	+	+
20	12.70	955.4524	[M − H]^−^	**955.4674**,835.4603, 793.4438	C_51_H_71_O_17/_C_44_H_50_O_26_	220; 450	Quercetin-3,4-diglucoside-3-(6-sinapoyl-glucoside)	+	+	+	+	+
21	13.77	793.3953	[M − H]^−^	**793.2434,** 310.2344, 169.5669	−	220	Unknown E	+	+	+	+	+
22	14.42	265.1488	[M − H]^−^	**265.7656,** 191.5755, 125.5444, 89.0097	−	220	Unknown F	+	+	+	+	+

+, present; **−,** not detected; and *confirmed using a pure chemical standard; **×**, unknown or no records in literature sources to our knowledge; literature sources and *standards were used to corroborate existing observations. MS^E^ fragments in boldtype face refers to the base peak (the highest peak); GA, gum-Arabic; MT, maltodextrin; control, non incapsulated.

#### 3.7.2. Quantification of some of the metabolites

Table 4 presents the quantitative analysis of phenolic acid (gallic acid) and flavonoids [(+)-catechin, (−)-epicatechin, and myricetin] in the samples. Gallic acid concentrations ranged from 33.62 μg/g DM (GA:MT, 1:1) to 44.83 μg/g DM (GA:MT, 0:1), with powders synthesized from MT alone exhibiting the highest concentration. The concentrations of (+)-catechin, (−)-epicatechin, and myricetin varied among the encapsulated powders. These compounds were significantly more abundant (*p* < 0.05) in BWEP produced using GA or MT alone than those produced from blended biopolymers. This could be due to differences in interaction mechanisms between these individual bioactive compounds and the carrier matrix, as each bioactive compound might interact differently with the encapsulating material due to polarity, molecular weight, and structure variations (58, 59). As a result, the blend of GA and MT might favor the encapsulation of a wider range of phenolic compounds, thereby increasing the total phenolic content (TPC), as reported earlier, while simultaneously reducing the concentration of certain specific phenolic compounds such as (+)-catechin, (−)-epicatechin, and myricetin in the combined biopolymers.

**Table 4:** Concentration of selected compounds in the BWEP encapsulated using different blending ratios of GA and MT.

Compound (μg/g DM)	GA:MT (1:0)	GA:MT (0:1)	GA:MT (1:1)	GA:MT (1:2)	GA:MT (2:1)
Gallic acid	34.77 ± 1.43^b^	44.83 ± 1.01^a^	33.62 ± 0.97^b^	34.87 ± 1.20^b^	34.59 ± 1.33 ^b^
(+)-Catechin	35.45 ± 0.85 ^a^	35.29 ± 1.62 ^a^	32.82 ± 2.07 ^b^	33.40 ± 0.33 ^b^	30.63 ± 0.31 ^b^
(−)-Epicatechin	45.89 ± 1.00 ^a^	40.55 ± 0.66^ab^	37.78 ± 0.59 ^b^	38.54 ± 1.32 ^b^	39.22 ± 0.33 ^b^
Myricetin	35.26 ± 1.22^a^	32.16 ± 1.52^ab^	31.41 ± 0.96 ^b^	30.07 ± 1.24 ^b^	30.59 ± 1.13 ^b^

Means (*n* = 3) in each row with different letter(s) (a–d) differ significantly (*p* < 0.05) according to Duncan’s multiple range test. GA, gum-Arabic; MT, maltodextrin.

## 4. Conclusion

The present study demonstrated the potential of GA and MT to preserve beetroot extract and prolong its storage life. The quality of the BWEP was dependent on the blending ratio. BWEP produced using GA or MT exhibited better color, solubility, encapsulation efficiency, and betalain content. Powders from the blends of GA and MT showed better oil holding capacity and total phenolic content. Meanwhile, quality attributes, including powder yield, total soluble solids, titratable acidity, bulk density, and DPPH radical scavenging activity, were not significantly (*p* > 0.05) affected by blending GA and MT. In comparison to the powders made from the blended biopolymers, BWEP produced using GA and MT individually was considerably smaller and more regular. Agglomeration was evident in all powders, although it was predominant in the powders made from the combined biopolymers. Metabolites were tentatively identified, including betalains, phenolic acids, and flavonoids. It was observed that the metabolites were associated with BWEP produced using GA and MT separately. The quantified metabolites, including gallic acid, (+)-catechin, (−)-epicatechin, and myricetin, were significantly higher in the BWEP produced from GA or MT separately, suggesting that the powders could be used to fortify other foods and formulate functional foods with specific health properties or as natural food colorants. Although blending GA and MT has the potential to improve the quality of BWEP, using these biopolymers separately showed a promise to promote a food circular bioeconomy. Future studies may focus on the storage stability and release kinetics of the produced powders.

## Data availability statement

The original contributions presented in the study are included in the article/supplementary material, further inquiries can be directed to the corresponding author.

## Author contributions

OF: conceptualization, resources, project administration, and funding acquisition. TM and OF: methodology. TK and OF: software, validation, and supervision. TM and TK: formal analysis. TM: investigation and writing—original draft preparation. TM, OF, and TK: writing—review and editing and visualization. All authors contributed to the article and approved the submitted version.

## Conflict of interest

The authors declare that the research was conducted in the absence of any commercial or financial relationships that could be construed as a potential conflict of interest.

## Publisher’s note

All claims expressed in this article are solely those of the authors and do not necessarily represent those of their affiliated organizations, or those of the publisher, the editors and the reviewers. Any product that may be evaluated in this article, or claim that may be made by its manufacturer, is not guaranteed or endorsed by the publisher.

## References

[1] Gustavsson J Cederberg C Sonesson U Van Otterdijk R Meybeck A. Global food losses and food waste Food and Agriculture Organization of the United Nations (2011) Rome, Italy.

[2] Nthimole CT Kaseke T Fawole OA. Micro-encapsulation and characterization of anthocyanin-rich raspberry juice powder for potential applications in the food industry. Processes. (2022) 10:1038. doi: 10.3390/pr10051038

[3] Banerjee J Singh R Vijayaraghavan R MacFarlane D Patti AF Arora A. Bioactives from fruit processing wastes: green approaches to valuable chemicals. Food Chem. (2017) 225:10–22. doi: 10.1016/j.foodchem.2016.12.093 28193402

[4] Vulić J Čanadanović-Brunet J Ćetković G Tumbas V Djilas S Četojević-Simin D. Antioxidant and cell growth activities of beet root pomace extracts. J Funct Foods. (2012) 4:670–8. doi: 10.1016/j.jff.2012.04.008

[5] Tumbas Šaponjac V Gironés-Vilaplana A Djilas S Mena P Ćetković G Moreno DA. Anthocyanin profiles and biological properties of caneberry (Rubus spp.) press residues. J Sci Food Agric. (2014) 94:2393–400. doi: 10.1002/jsfa.6564 24407975

[6] Tumbas Šaponjac V Gironés-Vilaplana A Djilas S Mena P Ćetković G Moreno DA. Chemical composition and potential bioactivity of strawberry pomace. RSC Adv. (2015) 5:5397–405. doi: 10.1039/c4ra14296a

[7] Chawla H Parle M Sharma K Yadav M. Beetroot: a health promoting functional food. Invent Rapid Nutraceut. (2016) 1:0976–3872.

[8] Mereddy R Chan A Fanning K Nirmal N Sultanbawa Y. Betalain rich functional extract with reduced salts and nitrate content from red beetroot (*Beta vulgaris* L.) using membrane separation technology. Food Chem. (2017) 215:311–7. doi: 10.1016/j.foodchem.2016.07.132 27542480

[9] Goulas V Manganaris GA. Exploring the phytochemical content and the antioxidant potential of Citrus fruits grown in Cyprus. Food Chem. (2012) 131:39–47. doi: 10.1016/j.foodchem.2011.08.007

[10] Jane S Vaishnav MMOP John S John S Monica SJ. All rights reserved. Int J Pharma Res Health Sci. (2017) 5:1974–9. doi: 10.12944/crnfsj.6.2.15

[11] Batoqui P Biondo F Boeing JS Oliveira Barizão É Evelazio De Souza N Matsushita M. Evaluation of beetroot (*Beta vulgaris* L.) leaves during its developmental stages: A chemical composition study. Food Sci Technol. (2014) 34:94–101. doi: 10.1590/s0101-20612014005000007

[12] Neha P Jain SK Jain NK Jain HK Mittal HK. Chemical and functional properties of beetroot (*Beta vulgaris* L.) for product development: a review. Int J Chem. (2018) 6:3190–4.

[13] Žitňanová I Ranostajová S Sobotová H Demelová D Pecháň I Ďuračková Z. Antioxidative activity of selected fruits and vegetables. Biologia. (2006) 61:279–84. doi: 10.2478/s11756-006-0051-7/html

[14] Dhiman A Suhag R Chauhan DS Thakur D Chhikara S Prabhakar PK. Status of beetroot processing and processed products: thermal and emerging technologies intervention. Trends Food Sci Technol. (2021) 114:443–58. doi: 10.1016/j.tifs.2021.05.042

[15] Čanadanović-Brunet JM Savatović SS Ćetković GS Vulić JJ Djilas SM Markov SL. Antioxidant and antimicrobial activities of beet root pomace extracts. Czech J Food Sci. (2011) 29:575–85. doi: 10.17221/210/2010-cjfs

[16] Chhikara N Kushwaha K Jaglan S Sharma P Panghal A. Nutritional, physicochemical, and functional quality of beetroot (*Beta vulgaris* L.) incorporated Asian noodles. Cereal Chem. (2019) 96:154–61. doi: 10.1002/cche.10126

[17] Champagne CP Fustier P. Microencapsulation for the improved delivery of bioactive compounds into foods. Curr Opin Biotechnol. (2007) 18:184–90. doi: 10.1016/j.copbio.2007.03.001 17368017

[18] Flores-Mancha MA Ruíz-Gutiérrez MG Sánchez-Vega R Santellano-Estrada E Chávez-Martínez A. Characterization of beet root extract (*Beta vulgaris*) encapsulated with maltodextrin and inulin. Molecules. (2020) 25:5498. doi: 10.3390/molecules25235498 33255296 PMC7727679

[19] Diaz YL Torres LS Serna JA Sotelo LI. Efecto de la Encapsulación en Secado por Atomización de Biocomponentes de Pitahaya Amarilla con Interés Funcional. Información Tecnológica. (2017) 28:23–34. doi: 10.4067/s0718-07642017000600004

[20] Whitaker S Guadarrama-Lezama A Cruz-Olivares J Martínez-Vargas S Carrillo-Navas H Román-Guerrero A. To establishing critical storage conditions of beetroot juice microcapsules by spray drying. Revista Mexicana de Ingeniería Química. (2014) 13:405–16.

[21] Serris GS Biliaderis CG. Degradation kinetics of beetroot pigment encapsulated in polymeric matrices. J Sci Food Agric. (2001) 81:691–700. doi: 10.1002/jsfa.864

[22] Ravichandran K Palaniraj R Saw NMMT Gabr AMM Ahmed AR Knorr D. Effects of different encapsulation agents and drying process on stability of betalains extract. J Food Sci Technol. (2014) 51:2216–21. doi: 10.1007/s13197-012-0728-6 25190886 PMC4152507

[23] Omae JM Goto PA Rodrigues LM Santos SS Paraiso CM. Beetroot extract encapsulated in inulin: storage stability and incorporation in sorbet. Cetjournal. (2017) 57:1843–8. doi: 10.3303/CET1757308

[24] Fang Z Bhandari B. Encapsulation of polyphenols – a review. Trends Food Sci Technol. (2010) 21:510–23. doi: 10.1016/j.tifs.2010.08.003

[25] Akbas E Kilercioglu M Onder ON Koker A Soyler B Oztop MH. Wheatgrass juice to wheat grass powder: encapsulation, physical and chemical characterization. J Funct Foods. (2017) 28:19–27. doi: 10.1016/j.jff.2016.11.010

[26] Jafari SM Assadpoor E He Y Bhandari B. Encapsulation efficiency of food Flavours and oils during spray drying. Dry Technol. (2008) 26:816–35. doi: 10.1080/07373930802135972

[27] Tolun A Altintas Z Artik N. Microencapsulation of grape polyphenols using maltodextrin and gum arabic as two alternative coating materials: development and characterization. J Biotechnol. (2016) 239:23–33. doi: 10.1016/j.jbiotec.2016.10.001 27720817

[28] Fang Z Bhandari B. Effect of spray drying and storage on the stability of bayberry polyphenols. Food Chem. (2011) 129:1139–47. doi: 10.1016/j.foodchem.2011.05.093 25212349

[29] Çam M Cihat N Erdo F. Pomegranate peel phenolics: microencapsulation, storage stability and potential ingredient for functional food development. Food Sci Technol. (2014) 55:117–23. doi: 10.1016/j.lwt.2013.09.011

[30] Sarabandi K Sadeghi Mahoonak A Hamishekar H Ghorbani M Jafari SM. Microencapsulation of casein hydrolysates: physicochemical, antioxidant and microstructure properties. J Food Eng. (2018) 237:86–95. doi: 10.1016/j.jfoodeng.2018.05.036

[31] Caliskan G Nur DS. The effects of the different drying conditions and the amounts of maltodextrin addition during spray drying of sumac extract. Food Bioprod Process. (2013) 91:539–48. doi: 10.1016/j.fbp.2013.06.004

[32] Mahdavee Khazaei K Jafari SM Ghorbani M Hemmati KA. Application of maltodextrin and gum Arabic in microencapsulation of saffron petal’s anthocyanins and evaluating their storage stability and color. Carbohydr Polym. (2014) 105:57–62. doi: 10.1016/j.carbpol.2014.01.042 24708952

[33] Jafari SM Ghalegi Ghalenoei M Dehnad D. Influence of spray drying on water solubility index, apparent density, and anthocyanin content of pomegranate juice powder. Powder Technol. (2017) 311:59–65. doi: 10.1016/j.powtec.2017.01.070

[34] Nadeem A Torun M Özdemir F. Spray drying of the mountain tea (Sideritis stricta) water extract by using different hydrocolloid carriers. Food Sci Technol. (2011) 44:1626–35. doi: 10.1016/j.lwt.2011.02.009

[35] Sarabandi K Peighambardoust SH Mahoonak AS Samaei SP. Effect of carrier types and compositions on the production yield, microstructure and physical characteristics of spray dried sour cherry juice concentrate. J Food Measure Character. (2017) 11:1602–12. doi: 10.1007/s11694-017-9540-3

[36] Adetoro AO Opara UL Fawole OA. Effect of carrier agents on the physicochemical and Technofunctional properties and antioxidant capacity of freeze-dried pomegranate juice (*Punica granatum*) powder. Foods. (2020) 9:1388. doi: 10.3390/foods9101388 33019645 PMC7600028

[37] Sarabandi K Jafari SM Mahoonak AS Mohammadi A. Application of gum Arabic and maltodextrin for encapsulation of eggplant peel extract as a natural antioxidant and color source. Int J Biol Macromol. (2019) 140:59–68. doi: 10.1016/j.ijbiomac.2019.08.133 31422189

[38] Magangana TP Makunga NP la Grange C Stander MA Fawole OA Opara UL. Blanching pre-treatment promotes high yields, bioactive compounds, antioxidants, enzyme inactivation and antibacterial activity of ‘wonderful’ pomegranate peel extracts at three different harvest maturities. Antioxidants. (2021) 10:1119. doi: 10.3390/antiox10071119 34356352 PMC8301009

[39] Fawole OA Makunga NP Opara UL. Antibacterial, antioxidant and tyrosinase-inhibition activities of pomegranate fruit peel methanolic extract. BMC Complement Altern Med. (2012) 12:1–11. doi: 10.1186/1472-6882-12-200 PMC3527236 23110485

[40] Wybraniec S Mizrahi Y. Fruit flesh Betacyanin pigments in Hylocereus Cacti. J Agric Food Chem. (2002) 50:6086–9. doi: 10.1021/jf020145k 12358484

[41] Dag D Kilercioglu M Oztop MH. Physical and chemical characteristics of encapsulated goldenberry (*Physalis peruviana* L.) juice powder. LWT. (2017) 83:86–94. doi: 10.1016/j.lwt.2017.05.007

[42] Bazaria B Kumar P. Effect of whey protein concentrate as drying aid and drying parameters on physicochemical and functional properties of spray dried beetroot juice concentrate. Food Biosci. (2016) 14:21–7. doi: 10.1016/j.fbio.2015.11.002

[43] Tonon RV Brabet C Pallet D Brat P Hubinger MD. Physicochemical and morphological characterization of açai (Euterpe oleraceae Mart.) powder produced with different carrier agents. Int J Food Sci Technol. (2009) 44:1950–8. doi: 10.1111/j.1365-2621.2009.02012.x

[44] Mohd Nawi N Muhamad II Mohd MA. The physicochemical properties of microwave-assisted encapsulated anthocyanins from *Ipomoea batatas* as affected by different wall materials. Food Sci Nutr. (2015) 3:91–9. doi: 10.1002/fsn3.132 25838887 PMC4376403

[45] George TT Oyenihi AB Rautenbach F Obilana AO. Characterization of *Moringa oleifera* leaf powder extract encapsulated in maltodextrin and/or gum Arabic coatings. Foods. (2021) 10:3044. doi: 10.3390/foods10123044 34945595 PMC8701997

[46] Seerangurayar T Manickavasagan A Al-Ismaili AM Al-Mulla YA. Effect of carrier agents on physicochemical properties of foam-mat freeze-dried date powder. Dry Technol. (2018) 36:1292–303. doi: 10.1080/07373937.2017.1400557

[47] Kobo GK Kaseke T Fawole OA. Micro-encapsulation of phytochemicals in passion fruit Peel waste generated on an organic farm: effect of carriers on the quality of encapsulated powders and potential for value-addition. Antioxidants. (2022) 11:1579. doi: 10.3390/antiox11081579 36009296 PMC9404774

[48] Barbosa J Borges S Amorim M Pereira MJ Oliveira A Pintado ME. Comparison of spray drying, freeze drying and convective hot air drying for the production of a probiotic orange powder. Function Foods. (2015) 17:340–51. doi: 10.1016/j.jff.2015.06.001

[49] Bazaria B Kumar P. Effect of dextrose equivalency of maltodextrin together with Arabic gum on properties of encapsulated beetroot juice. J Food Measure Character. (2017) 11:156–63. doi: 10.1007/s11694-016-9382-4

[50] Mohammed AN Ishwarya SP Nisha P. Nanoemulsion versus microemulsion Systems for the Encapsulation of beetroot extract: comparison of physicochemical characteristics and Betalain stability. Food Bioprocess Technol. (2021) 14:133–50. doi: 10.1007/s11947-020-02562-2

[51] Koç B Sakin-YIlmazer M Kaymak-Ertekin F BalkIr P. Physical properties of yoghurt powder produced by spray drying. J Food Sci Technol. (2014) 51:1377–83. doi: 10.1007/s13197-012-0653-8 24966433 PMC4062699

[52] Wang W Zhou W. Characterisation of spray dried soy sauce powders made by adding crystalline carbohydrates to drying carrier. Food Chem. (2015) 168:417–22. doi: 10.1016/j.foodchem.2014.07.065 25172729

[53] Cano-Chauca M Stringheta PC Ramos AM Cal-Vidal J. Effect of the carriers on the microstructure of mango powder obtained by spray drying and its functional characterization. Innovative Food Sci Emerg Technol. (2005) 6:420–8. doi: 10.1016/j.ifset.2005.05.003

[54] Dobroslavić E Elez Garofulić I Zorić Z Pedisić S Roje M Dragović-Uzelac V. Physicochemical properties, antioxidant capacity, and bioavailability of *Laurus nobilis* L. Leaf Polyphenolic Extracts Microencapsulated by Spray Drying. Foods. (2023) 12:1923. doi: 10.3390/foods12091923 37174461 PMC10177897

[55] Ramakrishnan Y Adzahan NM Yusof YA Muhammad K. Effect of wall materials on the spray drying efficiency, powder properties and stability of bioactive compounds in tamarillo juice microencapsulation. Powder Technol. (2018) 328:406–14. doi: 10.1016/j.powtec.2017.12.018

[56] Khalifa I Li M Mamet T Li C. Maltodextrin or gum Arabic with whey proteins as wall-material blends increased the stability and physiochemical characteristics of mulberry microparticles. Food Biosci. (2019) 31:100445. doi: 10.1016/j.fbio.2019.100445

[57] Lekshmi RGK Tejpal CS Anas KK Chatterjee NS Mathew S Ravishankar CN. Binary blend of maltodextrin and whey protein outperforms gum Arabic as superior wall material for squalene encapsulation. Food Hydrocoll. (2021) 121:106976. doi: 10.1016/j.foodhyd.2021.106976

[58] Fu Y Shi J Xie SY Zhang TY Soladoye OP Aluko RE. Red beetroot Betalains: perspectives on extraction, processing, and potential health benefits. J Agric Food Chem. (2020) 68:11595–611. doi: 10.1021/acs.jafc.0c04241 33040529

[59] Sansone F Picerno P Mencherini T Villecco F D’Ursi AM Aquino RP. Flavonoid microparticles by spray-drying: influence of enhancers of the dissolution rate on properties and stability. J Food Eng. (2011) 103:188–96. doi: 10.1016/j.jfoodeng.2010.10.015

